# Ambient Vibration Tests of an Arch Dam with Different Reservoir Water Levels: Experimental Results and Comparison with Finite Element Modelling

**DOI:** 10.1155/2014/692709

**Published:** 2014-06-05

**Authors:** Sergio Vincenzo Calcina, Laura Eltrudis, Luca Piroddi, Gaetano Ranieri

**Affiliations:** Department of Civil Engineering, Environmental Engineering and Architecture (DICAAR), University of Cagliari, via Marengo 2, 09123 Cagliari, Italy

## Abstract

This paper deals with the ambient vibration tests performed in an arch dam in two different working conditions in order to assess the effect produced by two different reservoir water levels on the structural vibration properties. The study consists of an experimental part and a numerical part. The experimental tests were carried out in two different periods of the year, at the beginning of autumn (October 2012) and at the end of winter (March 2013), respectively. The measurements were performed using a fast technique based on asynchronous records of microtremor time-series. In-contact single-station measurements were done by means of one single high resolution triaxial tromometer and two low-frequency seismometers, placed in different points of the structure. The Standard Spectral Ratio method has been used to evaluate the natural frequencies of vibration of the structure. A 3D finite element model of the arch dam-reservoir-foundation system has been developed to verify analytically determined vibration properties, such as natural frequencies and mode shapes, and their changes linked to water level with the experimental results.

## 1. Introduction

The knowledge of the structural response of buildings following the application of dynamic actions represents a key element to evaluate the buildings performance, especially in the case of extreme environmental conditions, mainly represented by earthquakes actions, or in the case of structures located near to the areas affected by mining activities (quarry explosions and other excavation activities) or underground constructions (e.g., tunnels and subways). In general, it is possible to observe that the dynamic behaviour of the structures is mainly influenced by the design features and by the geometry of the buildings and the interaction effects with the foundation soil and with other existing structures. Several authors show that the experimental dynamic response contributes to the assessment of structural or geotechnical damage identification and they use experimental vibration properties of the buildings for Structural Health Monitoring procedures implementation. Numerous papers are focused on the study of the behaviour of buildings in terms of seismic response of structures. These studies deal with the complex interaction phenomena between the foundation soil and the earthquake input (to assess the seismic performance of the buildings) and the seismic wave propagation (transmission and reflection) at the boundaries of the foundation level by means of both inertial and kinematic interaction [1]. Experimental dynamic behaviour plays an extremely important role for structures of public interest such as school buildings, hospitals, roads, and railway infrastructures (such as bridges and viaducts); structures of economic importance to land and water resources management; and for the electricity production as dams and artificial reservoirs. Furthermore, dynamic analysis methods of structures are required to assess the safety of existing structures and to evaluate proposed designs for the construction of new dams that are located in areas with significant seismicity. These studies are also performed to determine when structural modifications proposed are suitable and effective solutions to improve the seismic performance of old structures. The prediction of the actual vibration response of arch dams to dynamic loadings is a very complicated issue and depends on several factors including intensity and characteristics of the design input, the interaction of the dam with the foundation rock and reservoir water; the computational modelling technique used to simulate the behaviour of the structure; and the material properties used for the analysis. Main recommendations to perform correct dynamic analysis procedures are provided by Ghanaat [2]. In particular, the study of the dams dynamic properties involves the assessment of the interaction with rock foundation along the section and the water reservoir mass influence acting on the upstream side. The interaction problem between the dam body and the water impounded reservoir is an important factor affecting the dynamic response of arch dams when the ground shakes during an earthquake. Therefore this problem has been discussed by several authors. The first formulation has been proposed by Westergaard [3]. He simulated the effect of the water by means of the added mass concept, as a mass attached to the dam. A more appropriate representation of this concept is obtained using a finite element formulation able to describe the interaction and also for complicated geometry of the arch dam and the water reservoir [4]. However, both methods ignore the water compressibility and the energy losses caused by the radiation in the upstream direction and the reflections and refractions at the reservoir bottom of the pressure waves. These phenomena have been included in a more refined formulation [5]. These approaches require high computation efforts to determine frequency-dependent hydrodynamic pressure terms and to consider a range of reservoir-bottom reflection coefficients. Aminfar et al. [6] have developed the added-mass concept, first proposed by Westergaard [3]. These authors have observed that the interaction effects between an arch dam and the contained water-reservoir lead to an increase in the dam vibration periods, because the water moves with the dam increasing the total mass that is in motion. Furthermore the added water mass can determine the partial absorption of pressure waves at the reservoir boundaries and this fact may be the fundamental cause of an increase in structural damping properties. In other works the proposed model highlights the earthquake response change of the dam and this behaviour as depending on the mass of the impounded water inside the reservoir. Sevim et al. [7] have investigated the water level effects on the dynamic response of the dams by means of ambient vibration testing on a prototype arch dam reservoir-foundation model performed in a laboratory. These authors observe that the difference between the first natural frequencies measured with an empty reservoir and with a full reservoir ranges from 20% to 25%. Analytical models have often been developed in order to get the dynamic characteristics of the dams or to assess the correlation between experimental dynamic response (obtained by means of measurements of displacements, velocities, or accelerations acquired on the structure body) and numerical simulations [8, 9]. Generally an elastic or failure physical model may be used, depending on the purposes of the research. The identification of natural frequencies and the estimation of modal shapes and structural damping can be achieved by means of numerical simulations based on an elastic physical model. Conversely, in order to study the structures behaviour to strong actions (e.g., seismic actions), we must use more complex failure physical models able to reproduce the possible opening of joints and cracking of the concrete dams [10]. Other authors have used continuous ambient vibrations recordings to monitor the effects of the varying water level throughout the testing period [11].

This paper aims to assess the influence of different water level heights on dynamic properties on a double curvature arch dam real structure by means of in situ experimental ambient vibration tests carried out with one single-station high sensitive triaxial tromometer and with two short-period seismological seismometers. The stored traces have been compared and processed in order to perform spectral, short-time, and directional Fourier analyses. The microtremor time-series are analysed using Standard Spectral Ratio method [12] to enhance the spectral components related to the dynamic response of the dam. In this work spectral ratios are calculated, respectively, for radial, tangential, and vertical components of the motion. In order to attempt an explanation for the different behaviour achieved by the experimental surveys in both conditions, a dynamic numerical analysis of the dam was done using a 3D finite element model of the system composed by structure-foundation rock-reservoir, obtained imposing different reservoir water levels.

## 2. The Site of Study

**Figure 1:**
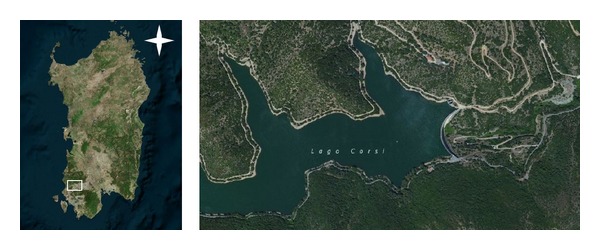
Location of Punta Gennarta Dam and satellite image of the water reservoir Corsi Lake.

**Figure 2:**
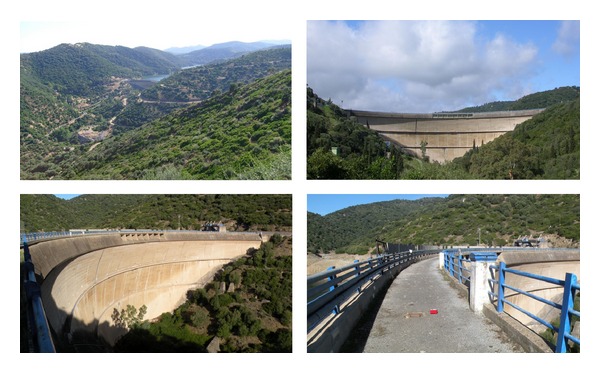
Some pictures of Punta Gennarta Arch Dam.

The structure of Punta Gennarta Dam is a Reinforced Concrete variable radius arch dam located on the Canonica river in South-West Sardinia, Italy (as shown in Figure 1), at the northern side of Iglesias town. The actual structure was built over four years (1959–1962) using about 58,600 m^3^ concrete for its body. It retains about 12.70 · 10^6^ m^3^ reservoir water (named Corsi Lake). The structure is characterized by variable radius and angles that provide an asymmetrical geometry along crown cantilever. It is 57 metres in height (above foundation) and has 254 metres crest length. Its crest length to height ratio is 4.46. The thickness of the crown cantilever ranges between 2.30 metres at the crest and 9.90 metres at the base. It is provided with two galleries inside its body. The first one is located in the upper part and is used to keeping the measurement equipment such as strainmeters, jointmeters, extensometers, and other monitoring tools. The second tunnel is placed in the lower part of the structure and it is accessible through a narrow central opening. Some pictures of Punta Gennarta Arch Dam are shown in Figure 2. Other geometric features are summarized in Table 1. The site of the structure is classified in Class IV of the Italian seismic zonation, corresponding at very low seismic hazard areas. The dam is built on a weak, narrow valley characterized by geological metamorphic formations mainly consisting of metasandstones (lower Cambrian). The characteristics of easy accessibility of the site and the possibility of installing sensors into small niches opening on the downstream side of the dam are suitable for the experimental surveys and to obtain vibration data distributed all along the structure body.

## 3. Experimental Vibration Tests and Results

### 3.1. Description of Data Acquisition and Processing

**Figure 3:**
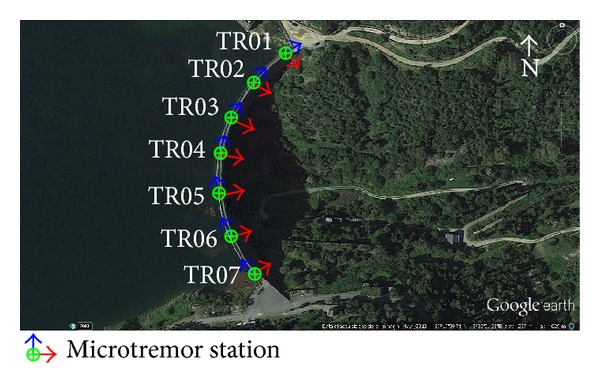
In-contact sensor stations placed on the dam crest.

**Figure 4:**
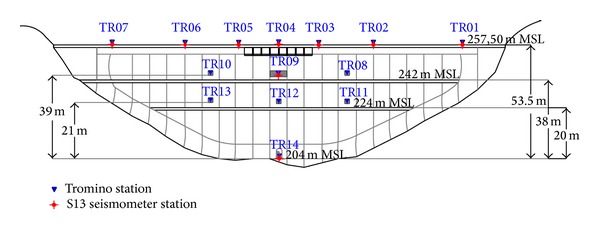
In-contact microtremor stations placed at different floors of Punta Gennarta Arch Dam, downstream side view (blue triangles indicate Tromino stations; red circles mark S13 stations).

The experimental dynamic characterisation of Punta Gennarta Dam was conducted only with passive survey mode through ambient vibration measurements, in agreement with the widespread NIMA techniques (Noise Input Modal Analyses). Ambient vibration tests represent an effective, fast, and relatively economical method of estimating fundamental dynamic features of the structures. In the last years several authors have proposed different methods that aimed to improve passive dynamic characterization techniques of structures using records of natural microtremor [13–17]. The different number of sensors used to configure the experimental layout (from a single triaxial sensor to a multiple sensors network) affects the information degree that we can obtain by the surveys [18]. During this experiment one digital high resolution tromograph Tromino has been used for ambient vibration record. This tridirectional sensor was oriented with one horizontal axis in the same direction of the dam local curvature radius. In other words, for each acquisition station, the displacement field was acquired along three components, respectively, radial, tangential, and vertical component. The acquisition setup is composed by fourteen asynchronous ambient vibration recording stations placed on the dam body. The distribution of the measurement points on the structure is shown in Figure 3 for seven stations placed on the crest level and in Figure 4 for all stations (frontal view of the downstream side of the structure). The measurements were performed in a relatively short time (about two hours) in order to ensure the condition of maximum stationarity of the noise seismic field. Ten-minute time-series were sampled with 0.002 seconds sample interval. Only at the topmost of the dam longer microtremor time-series (30 minutes) were acquired to enhance structural damping estimation procedures. Ambient vibration tests were repeated using the same acquisition geometry with two dam operational conditions characterized by different water reservoir levels. In both cases, the modal parameters obtained were natural frequencies, mode shapes, and damping ratios. The first measurement campaign was carried out in October 2012 with about 27-metre (231 m s.l.m) water level height. The next measurements were collected in March 2013 with 43-metre (247 m s.l.m) water level height. In order to compare the achieved experimental results, the acquisitions were done with similar weather conditions. Before performing the spectral analysis the signals were equalized, padded, detrended, and tapered with Bartlett window to reducing leakage effects. Natural frequencies of vibration were extracted by means of the Horizontal to Horizontal Spectral Ratio method (HHSR). Empirical dynamic response in radial, tangential, and vertical, respectively, direction assumes the following expression:

(1) $$\begin{array}{c} {\text{SSR}}_{\text{rad}}\left(\omega \right)=\frac{\left|H_{i}\left(\omega \right)\right|}{\left|H_{\text{ref}}\left(\omega \right)\right|}, \end{array}$$

where |*H*_*i*_(*ω*)| indicates the microtremor amplitude spectrum of the *i*th measurement station and |*H*_ref_(*ω*)| the amplitude spectrum of the [TR14] station, placed inside the lower tunnel and used as reference site. Its amplitude spectrum represents the inverse filter able to highlight the experimental dynamic characteristics of the dam. In fact looking at the spectral ratios in time domain, starting from the stationary linear systems theory, for each component of motion, the output signal on the top floor of the structure is in this way deconvolved with the input signal acquired at the ground floor of the structure, leading to the estimation of its empirical transfer function. This technique, developed in seismological context [12], has been effectively applied to perform experimental dynamic analyses of civil structures such as residential buildings and towers using microtremor time-series and appears to be a reliable method for assessing natural frequencies [14, 15]. Due to the higher sensitivity of the velocity transducers, velocity traces were used for SSR analysis. To estimate the damping ratio at the fundamental frequency of the structure a simplified nonparametric method proposed by Mucciarelli and Gallipoli [19] was applied. This technique is based on the general assumption that the first approximation of the dynamic behaviour of the engineering structures is provided by the Single-Degree-Of-Freedom oscillator model. This approach allows us to assess with good approximation the damping ratio associated with the first mode shape by means of only a ten-minute recording of ambient vibration acquired at the topmost part of a building. To compare the experimental results obtained using Tromino with the results achieved by other measurement tools, we have also used two high resolution short-period seismometers (Teledyne Geotech Model S13). These moving coil electromagnetic velocity-transducers are mainly designed for geophysics research and are capable of meeting the noise and stability requirements of the most exacting studies. Their operation condition is convertible in both vertical and horizontal modes and, depending on the purposes of the survey, their natural frequency can be adjusted between 0.75 Hz and 1.1 Hz. To perform this study, one sensor has been configured to acquire the horizontal component of motion (along the radial direction of the dam), whereas another geophone has been used in vertical operation mode. Both horizontal and vertical signals were recorded by one Single Geode seismograph unit (Geometrics). For each station we have acquired twenty time-histories of 32 s (maximum time acquisition length with 2 ms sample interval). The seg2 files are next merged and converted in ASCII format to be analysed in the Matlab environment. The relatively bulky size of the S13 sensors does not allow the placement of the instrumental tool at some station points, in particular for three stations located at the lower walkway level and for those to the left and right of the upper walkway. All S13 microtremor stations are marked with red circles in Figure 4.

### 3.2. Tests Results and Discussion

**Figure 5:**
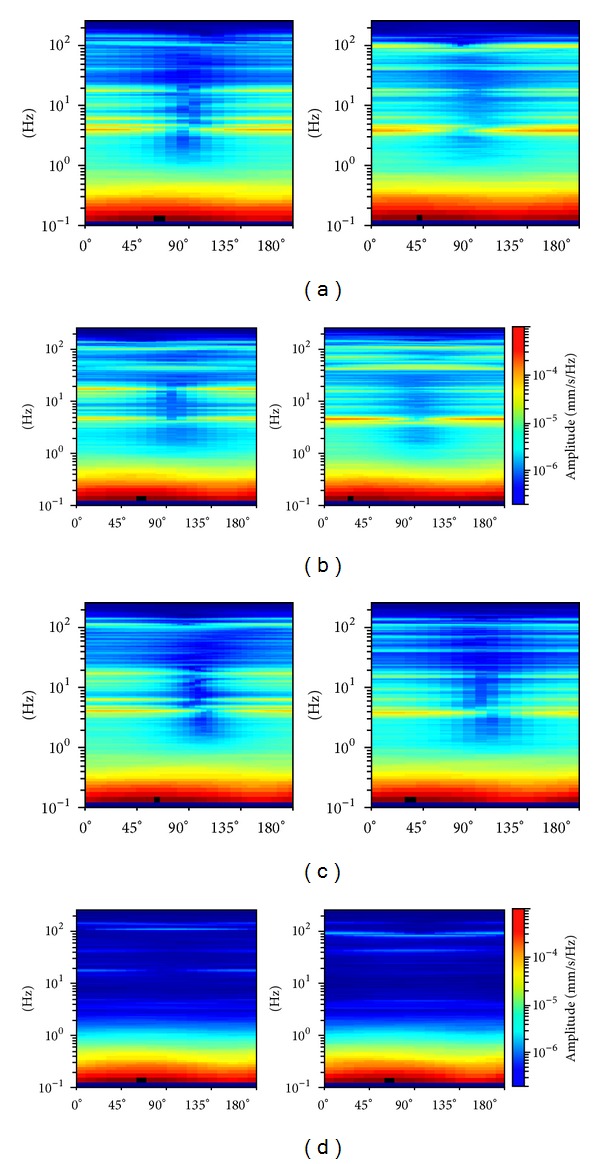
Directional spectra derived for the TR03 (a), TR04 (b), TR05 (c), and TR14 (d) microtremor horizontal traces acquired during both passive surveys.

**Figure 6:**
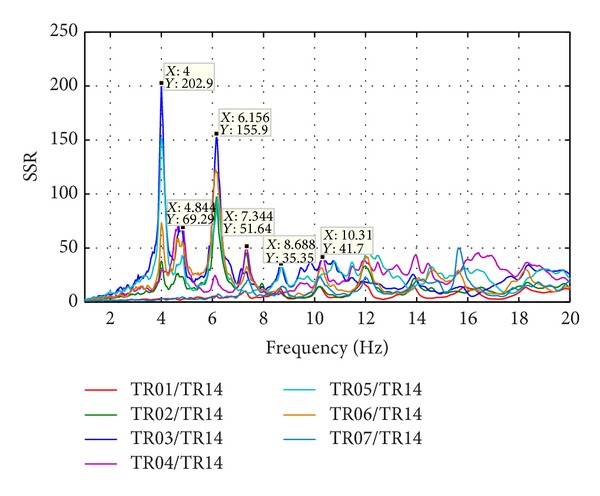
Radial spectral ratios at the dam crest (October 2012).

**Figure 7:**
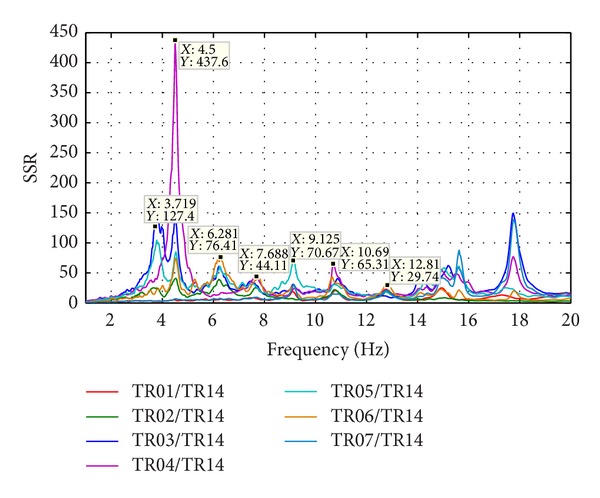
Radial spectral ratios at the dam crest (March 2013).

**Figure 8:**
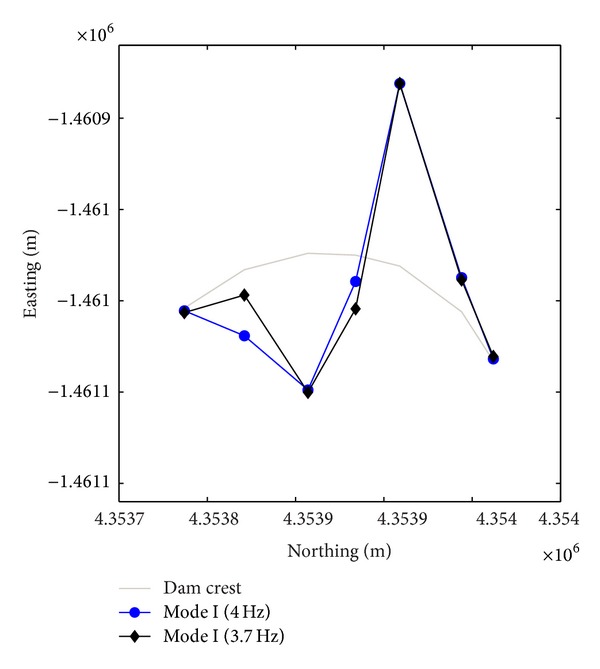
Normalised crest plot of the empirical first mode shape derived by the ambient vibration surveys with 27-metre water level (4 Hz) and 43-metre water level (3.7 Hz).

Natural frequencies are highlighted by amplitude peaks in both radial and tangential horizontal spectra of the recorded time-histories at several floors of the Punta Gennarta Dam. Amplitude spectra used for this analysis were derived only by velocity time-histories for the higher sensitivity and very low instrumental self-noise of the velocimeter transducers. Spectral analyses have been performed only selecting the time intervals not affected by transients using Gabor's transform. All amplitude spectral ratios were obtained by filtering the amplitude spectrum of each microtremor trace with the reference amplitude spectrum computed for the signal acquired at [TR14] station, collected above the foundation level of the dam, within the lower tunnel. Directional spectral analysis of all microtremor data puts in evidence that radial displacements represent the main vibration components of the arch dam motion. Thus, the traces acquired in this direction can be used to effectively evaluate the empirical transfer function of the dam. To demonstrate this fact we can observe Figure 5 where the directional spectra of the horizontal components for some records are compared. Microtremor signals acquired at the topmost part of the structure are characterized by a strong directional effect of the main spectral components (corresponding to the natural frequencies of the dam). In these plots, 0° and 90° indicate radial and tangential directions, respectively. Directional effects are progressively attenuated towards the lower levels of the structure until the base station, where the [TR14] angular plot does not show any anisotropic features of the noise field. Radial spectral ratios derived for all microtremor stations placed on the crest of the dam are included in Figure 6 and in Figure 7. As we can observe, results obtained in October 2012 highlight the fundamental frequency of vibration at about 4 Hz considering the radial component of the motion. The peak related to the fundamental mode is characterized by maximum amplitude in correspondence with the [TR03] and [TR05] microtremor stations. Conversely, this frequency shows negligible amplitude in the spectral ratio derived from the [TR04] station placed in the central position at the crest of the dam, as can be seen in both figures. The amplitude of this frequency peak is strongly attenuated in the signals acquired at stations [TR01] and [TR07], measurements performed near to the abutments of the dam. These records are not affected by the main vibration frequencies of the structure; rather their spectra are characterized only by frequency peaks above 7 Hz, related to structural higher modes of vibration. The absence of the vibration component with frequency of 4 Hz in the [TR04] recording allows us to assume that the first vibration mode shape of the structure is not represented by a simple flexural mode but is characterized by one node in the central part of the dam. This hypothesis is also supported by the characteristics of the spectral ratios relative to the measurements taken in both upper and lower walkways, where the spectral peak associated with the first vibration mode shows larger amplitude in the side positions of the structure rather than in two central positions, where it is negligible or missing (like for the [TR09] and [TR12] stations). Furthermore, the spectral ratios show a clear peak centered at the frequency of 4.8 Hz. This component could be associated with the second mode of vibration of the structure. In this case the mode shape retrieved at 4.8 Hz is not provided by one node in correspondence of the central acquisition station [TR04], where the amplitude of this component is at its maximum. An additional spectral peak at the frequency of about 6.2 Hz is probably related to a higher vibration mode. The frequency range between 7 Hz and 20 Hz is instead characterized by other frequency peaks of further vibration structural modes of the dam. The measurements carried out in March 2013 supply evidence that the first frequency of vibration is now about 3.7 Hz. The frequency shift from 4 Hz to 3.7 Hz between the conditions of two reservoir water levels corresponds with a reduction of about 7.5%. Because the structure features were not modified during the two surveys (few months), this variation is directly related to different water pressure acting on the upstream side of the dam. The second frequency of vibration is 6.25% lower than the value measured in October 2012, reducing from 4.8 Hz to 4.5 Hz. Higher frequencies of vibration are either not clearly affected by this effect, or the observed variation is negligible. The first experimental mode shape of vibration for the crest arch of the dam is shown in Figure 8. In both cases we can observe an antisymmetric fundamental mode shape with maximum displacements at the [TR03] and [TR05] microtremor stations. Several empirical relationships proposed in literature can be used to assess fundamental frequency of arch dams by the geometric features of these structures. In particular, Priscu et al. [20] present the natural fundamental frequency of arch dams corresponding at the full reservoir condition by means of the following equation:

(2) $$\begin{array}{c} f_{0}=\frac{1}{0.1+0.2\left(H/100\right)}, \end{array}$$

where *f*_0_ indicates the natural fundamental frequency and *H* (in metres) is the dam height. The natural frequencies of Punta Gennarta Dam, obtained from the two ambient vibration tests, are summarized in Tables 2 and 3. As can be seen in these tables, the natural frequencies are close to each other. We have compared experimental vibration properties with expected values using the above empirical formulation. According to (2), the natural fundamental frequency of Punta Gennarta Dam is 4.6 Hz while the reservoir is full. Whereas the natural fundamental frequency obtained from ambient vibration test is 3.7 Hz. However, this value is not obtained for full reservoir condition, but for the highest water level during the analysed periods (corresponding to about 83.8% of the maximum reservoir height). Furthermore, the above equation does not account for the aspect ratio of the dam, completely neglecting both the crest length and the thickness of the structure. Therefore, this kind of difference may be accepted. This result shows that the reservoir water interaction phenomena have more importance on the dynamic behaviour of these structures.

**Figure 9:**
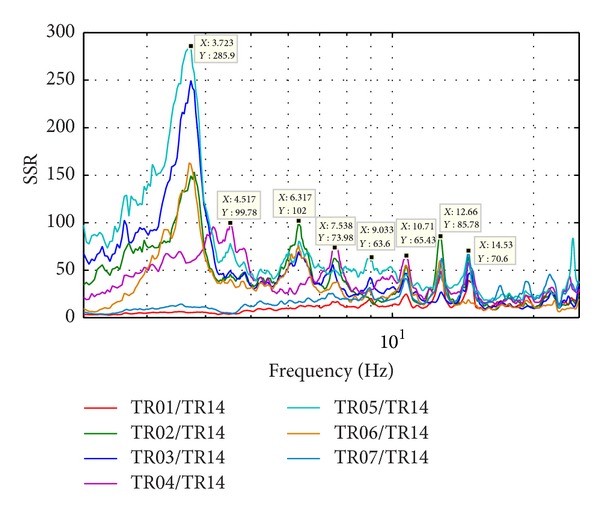
Amplitude spectral ratios derived for the crest stations using S13 seismometers time-recordings with 43-metre water level height.

**Figure 10:**
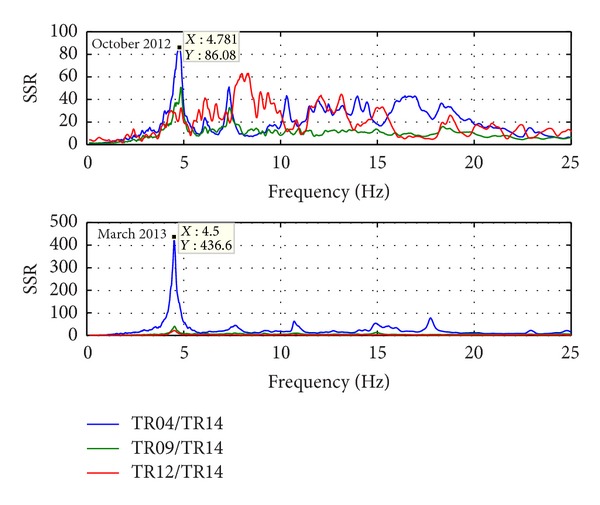
Empirical transfer function derived for the crown cantilever vertical section during both ambient vibration tests.

**Figure 11:**
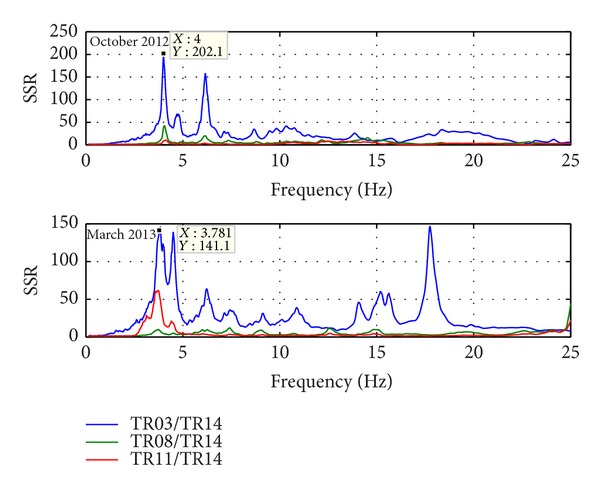
Radial spectral ratios derived for the left side of the dam.

**Figure 12:**
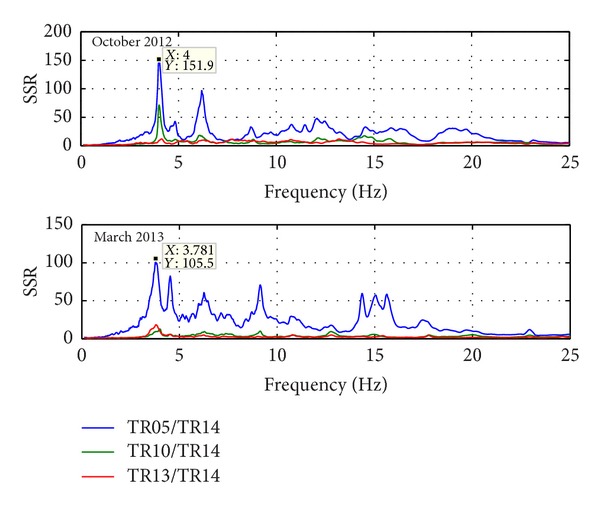
Radial spectral ratios derived for the right side of the dam.

**Figure 13:**
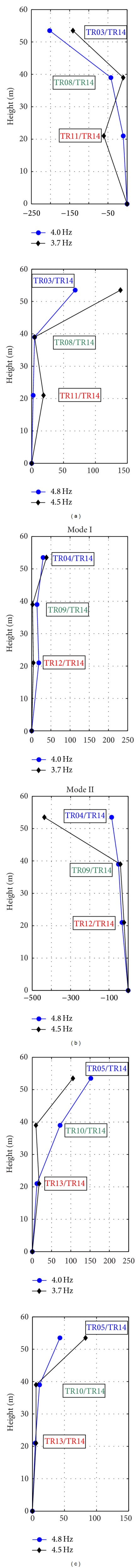
Comparison between the experimental mode shapes evaluated in terms of relative amplification effect, derived for: (a) the left side of the dam; (b) the crown cantilever vertical section; (c) the right side of the dam.

**Figure 14:**
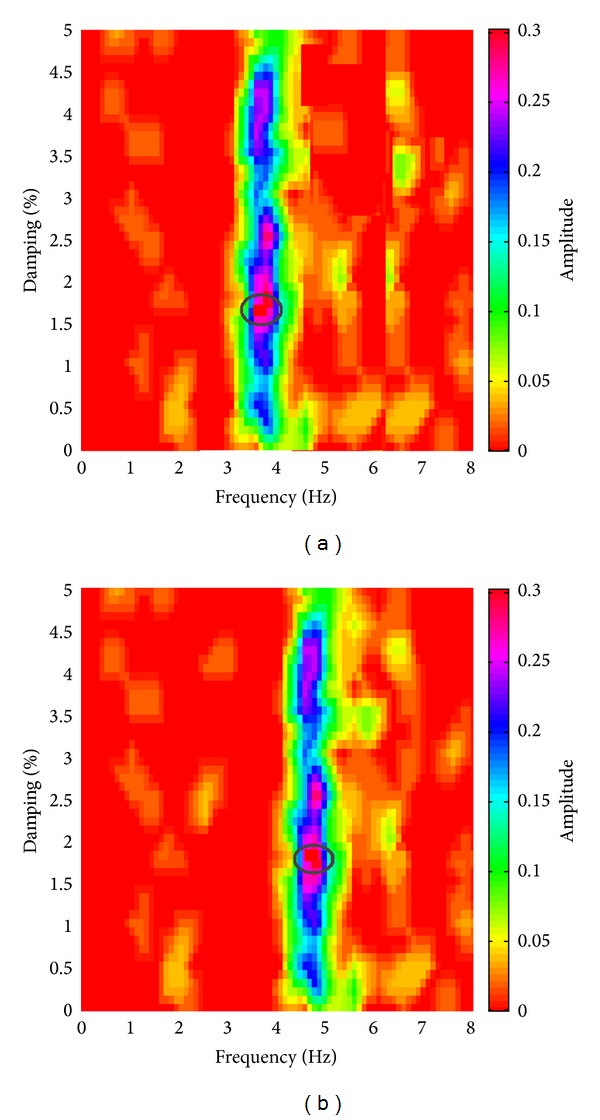
Example of NonPaDAn damping estimation performed for the first (a) and second (b) vibration modes of the dam. Maximum values, indicated by means of a black ellipse, correspond to each natural frequency and to the related modal damping.

Further evidence of the experimental results obtained from the recordings acquired with the digital tromograph Tromino comes from the comparison with the microtremor spectra recorded through the S13 Teledyne seismometers. In fact, it is possible to detect the same frequency components using both instrumental tools, as we can see in Figure 9, where radial spectral ratios related to the crest stations acquired using S13 geophones are shown. By observing these spectra it is possible to note that all frequency peaks are very close to those identified using the other sensor. Only the frequency of the second vibration mode shows smaller amplitudes, except for the midpoint crest station [TR04]. The water level variation effect is also highlighted by the different behaviour of several points of the structure body during two test conditions. The experimental mode shapes are obtained by comparing the measurements acquired along four floors of the structure, above the foundation level (taken as reference point at 0 metres), at the lower and at the higher walkways (21 and 39 metres), and at the crest of dam (53.5 metres), respectively. Radial spectral ratios derived for these points are used to assess the relative amplification effect of vibration produced from the base to the top of the dam, along its crown cantilever section (Figure 10) and two side sections, at the left (Figure 11) and at the right (Figure 12) of the structure. The relative amplitudes have been compared to measurements collected on different days, although with analogous environmental and weather conditions. The comparison is shown in Figure 13 where we can see that during the test carried out with the lower water level (27 metres) all considered points of the dam are characterized by an amplification effect, progressively increasing from the base to the crest for both first and second natural frequencies. Conversely, results obtained in the next ambient vibration test (with 43-metre water level height) highlight a deviation from the previous behaviour and we can observe that there is a greater amplification of the motion at the lower walkway level. This behaviour can be observed in both sides of the structure for the fundamental frequency of vibration, but it is more marked in the left side of the dam. Along the crown cantilever section we can observe that the central section of the dam is mainly affected by the second vibration mode (at the frequency of 4.8 Hz in October 2012 and 4.5 Hz in March 2013). Moreover the experimental dynamic behaviour of the dam associated with higher water level is characterised by greater amplification at the dam crest rather than the case of lower water level. Damping ratios derived for the first and second vibration modes of the dam are also summarized in Tables 2 and 3. These values have been obtained by means of the matrices shown in Figure 14. In these plots, maximum amplitudes indicate the percentage of modal damping related to each natural frequency of vibration detected. However, in contrast to natural frequencies, damping ratios do not show a significant change with two different water levels.

## 4. Finite Element Modelling and Analysis

### 4.1. Discrete Model of Punta Gennarta Dam

**Figure 15:**
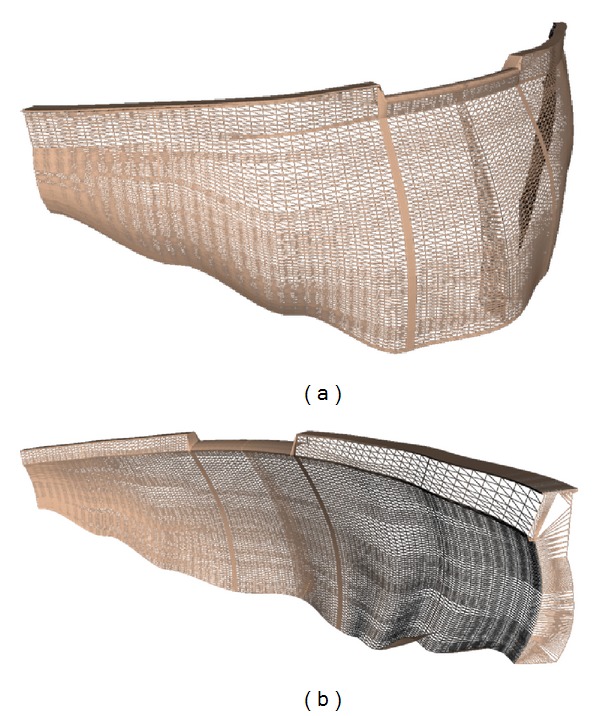
Some pictures of the 3D mesh used for the finite element analysis of Punta Gennarta Dam (a) upstream side view; (b) downstream side view.

In order to explain whether the differences observed in the fundamental frequencies are really related to variation in the water level reservoir a numerical dynamic analysis was carried out, taking into account the foundation rock-structure-reservoir interaction. 3D Structural Mechanics Module (eigenfrequency analysis solver) of Comsol Multiphysics package was used to calculate the mode shapes and the undamped natural frequencies. The finite element model of the Punta Gennarta Dam is built with variable radius curvature geometry obtained through the interpolation of seven vertical sections of the structure in 3D space. In fact, all authors highlight that arch dam structures must be modelled as three-dimensional objects to obtain a realistic representation of their structural behaviour. The discrete model used to predict dynamic vibration properties does not consider the openings of the structure. The mesh size is established in agreement with literature guidelines [21]. The dam body is built by means of 4,734 tetrahedral 3D elements. These elements compose the base mesh of the discrete model and each node of the model has three degrees-of-freedom, corresponding to translations in *x*, *y*, and *z* directions. The total number of degrees-of-freedom amounts to 26,799 for the extended mesh. In the finite element model the compatibility and equilibrium conditions are automatically satisfied at the nodes along the interfaces between Dam-Reservoir-Foundation because the displacements are assumed as variables in both the reservoir and the dam, following classic Lagrangian approach. Domain properties defined for the analysis are density, elasticity modulus and Poisson's ratio. The density was taken to be 2,300 kg/m^3^, the Young's modulus 25E09 N/m^2^, and Poisson's ratio was assumed to be 0.33. Some views of the finite element model of the Punta Gennarta arch dam are shown in Figure 15.

### 4.2. Numerical Results

**Figure 16:**
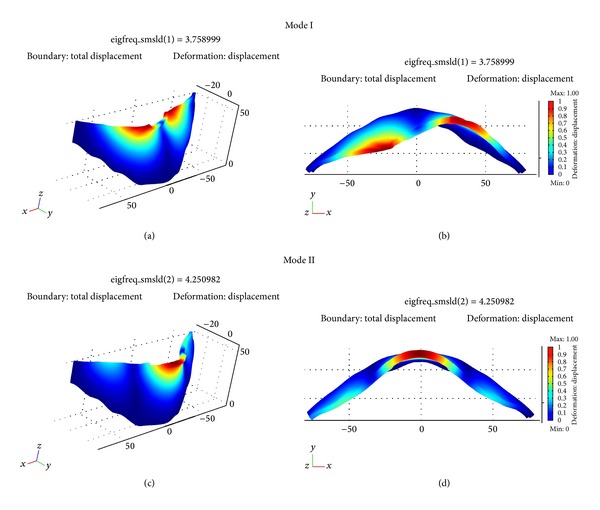
First two mode shapes of vibration according to Punta Gennarta Dam model: (a, c) perspective view; (b, d) horizontal plane view. The color scale indicates the normalized total displacement of each mode shape.

The Finite Element code solves the generalized eigenvalues problem to describe free undamped vibrations of a finite element linearly elastic system. This problem is defined by the following equation:

(3) $$\begin{array}{c} \left(K-\lambda_{n}M\right)\Theta_{n}=0\;n=1,\ldots ,N, \end{array}$$

where *K* and *M* are the stiffness matrix and the mass matrix of the structure, respectively, and *N* represents the total number of degrees-of-freedom of the discrete model. The solution leads to define *N* eigenvalues *λ*_*n*_ and the corresponding eigenvectors Θ_*n*_. Eigenvalues are related to natural frequencies (*ω*_*n*_ = *λ*_*n*_^1/2^) and each eigenvector describes one single mode shape. The mode shapes obtained for this model are plotted in Figure 16. This numerical model allows us to predict the vibration properties of the dam with both considered water levels. Water level effect has been simulated by means of different boundary conditions on the upstream surface of the structure finite element model. In particular, at the nodes of the mesh placed along the contact surface with the water reservoir only vertical displacements are allowed. In other words, by taking into account the water pressure acting on the upstream surface of the structure, we have built the finite element model approximating the curvature of the upstream surface with several faces characterized by variable curvature and fixed height. In this way, we have imposed only vertical displacement boundary condition at the nodes placed along the faces in contact with the water reservoir. Conversely, the free boundary condition has been assumed for the faces placed above the water level table. The interaction between the dam structure and the rock foundation corresponds with the fixed nodes boundary condition (zero displacements and rotations along the interface between structure and rock foundation). The mode shapes can be generally classified into symmetrical, antisymmetrical, and vertical modes, respectively. We can see that the fundamental mode shape of the structure is an antisymmetrical vibration mode and it shows a node at the midway point of the dam crest, corresponding about to the [TR04] microtremor station position. There are no displacements along the crown cantilever section for this mode shape. The second vibration mode is characterized by a more complicated shape with maximum displacement localised in the upper part of the crown cantilever section. Thus, the second mode is classified as a symmetric mode shape. The first natural frequency of the dam model results in 4.03 Hz and 3.75 Hz considering the different water level height. These values are very similar to those derived by vibration spectra. Conversely, there is a small difference for the second natural frequency of the finite element model with results of 4.56 Hz and 4.25 Hz, respectively, therefore in both cases lower than those experimentally measured. This fact could be related to more complex interaction phenomena not fully modelled through Finite Element analysis or to assigned material properties, not perfectly matching true values. These limitations could be probably overcome also by improving numerical simulation of the fluid-structure interaction effects using more complex multiphysics models. However, for the purposes of this research, despite the above small differences, we can consider that numerical results confirm the experimental dynamic behaviour of the dam and the observed frequency shift following the changes of the water height.

## 5. Conclusions

Through the passive survey method we have derived the main vibration properties of a double curvature arch dam by considering two different water levels. Experimental vibration analyses carried out by means of a simple instrumental layout (composed of a single tridirectional tromometer and two short period seismometers) have allowed us to observe the variation of the first two natural frequencies of the dam related to the effect produced by the impounded water within the artificial reservoir. Fourteen microtremor recording stations have been chosen above the structure on the downstream surface. Natural frequencies are derived using Standard Spectral Ratio method modified for the purposes of this research into radial, tangential, and vertical spectral ratio, respectively. The radial component of the motion is that mainly affected by the vibration properties of the dam and it has been used for eigenfrequency peak picking. Observed frequency shift for the first vibration mode results 7.5% passing from 4 Hz to 3.7 Hz. Also the second mode shows the same variation (about 6.25% from 4.8 Hz to 4.5 Hz). Higher modes do not appear significantly influenced by this effect. Furthermore, although we have acquired only asynchronous time-histories, the measurements have allowed us to define the shape of the fundamental vibration mode. In order to validate experimental evidences, a numerical dynamic analysis has been performed by finite element modelling of the structure. This numerical model has allowed us to observe the water interaction effect imposing several boundary conditions at the nodes of the 3D mesh along the interface between the upstream side and water volume stored within the reservoir. The obtained results have shown a good agreement with vibration data and have confirmed the hypothesis about the shape of the fundamental vibration mode.

## Figures and Tables

**Table 1:** Geometric features of Punta Gennarta Arch Dam.

Description	metres s.l.m.
Max. height of reservoir water level	255.30
Shallow spillway altitude	249.00
Bottom spillway altitude	204.50
Lower walkway altitude	224.00
Upper walkway altitude	242.00
Crest altitude	257.50

**Table 2:** Experimental vibration properties of Punta Gennarta Dam—October 2012.

Mode number	Frequency *f* (Hz)	Damping ratio *ξ* (%)
1	4.0	1.64
2	4.8	1.91
3	6.2	—
4	7.3	—

**Table 3:** Experimental vibration properties of Punta Gennarta Dam—March 2013.

Mode number	Frequency *f* (Hz)	Damping ratio *ξ* (%)
1	3.7	1.73
2	4.5	1.82
3	6.3	—
4	7.5–7.7	—
